# Supplementary material for: VEGFA-Positive Macrophages Regulate Aqueous Humor Outflow in Aged Mice and Humans
Source: bioRxiv. 2026 Sep 11:2026.08.20.746006. Preprint. [Version 3] doi: 10.64898/2026.08.20.746006 (PMC13622583; doi:10.64898/2026.08.20.746006)
Supplement: Supplementary file 1 [file media-1.pdf]

SUPPLEMENTAL MATERIALS

VEGFA-Positive Macrophages Regulate Aqueous Humor Outflow in Aged Mice and Humans

Naoki Kiyota[1,2], Yalu Zhou[1,2], Dilip K. Deb[1,2], Guohui Ren[1,2], Tuncer Onay[1,2], Ester Reina-Torres[3], Hoi-Lam Li[4], Constance E. Runyan[5], Robert S. Feder[6], Hyunjoo J. Lee[7], Darryl R. Overby[3], Haiyan Gong[4], G. R. Scott Budinger[5], Benjamin R. Thomson[1,6,8], and Susan E. Quaggin[1,2]

[1] Feinberg Cardiovascular and Renal Research Institute, Northwestern University Feinberg School of Medicine, Chicago.

[2] Department of Medicine, Northwestern University Feinberg School of Medicine, Chicago, IL.

[3] Department of Bioengineering, Imperial College London, London, UK.

[4] Department of Ophthalmology and Department of Anatomy and Neurobiology, Boston University Chobanian & Avedisian School of Medicine, Boston, MA.

[5] Division of Pulmonary and Critical Care Medicine, Department of Medicine, Northwestern University Feinberg School of Medicine, Chicago, IL.

[6] Department of Ophthalmology, Northwestern University Feinberg School of Medicine, Chicago, IL.

[7] Department of Ophthalmology, Harvard Medical School, and Massachusetts Eye and Ear, Boston, MA.

[8] Department of Biomedical Engineering, Northwestern University McCormick School of Engineering, Evanston, IL.

Correspondence to: Susan E. Quaggin, MD

676 N. St. Clair Ave., Suite 2300, Chicago, IL 60611, USA.

Email: quaggin@northwestern.edu

Supplementary Figures

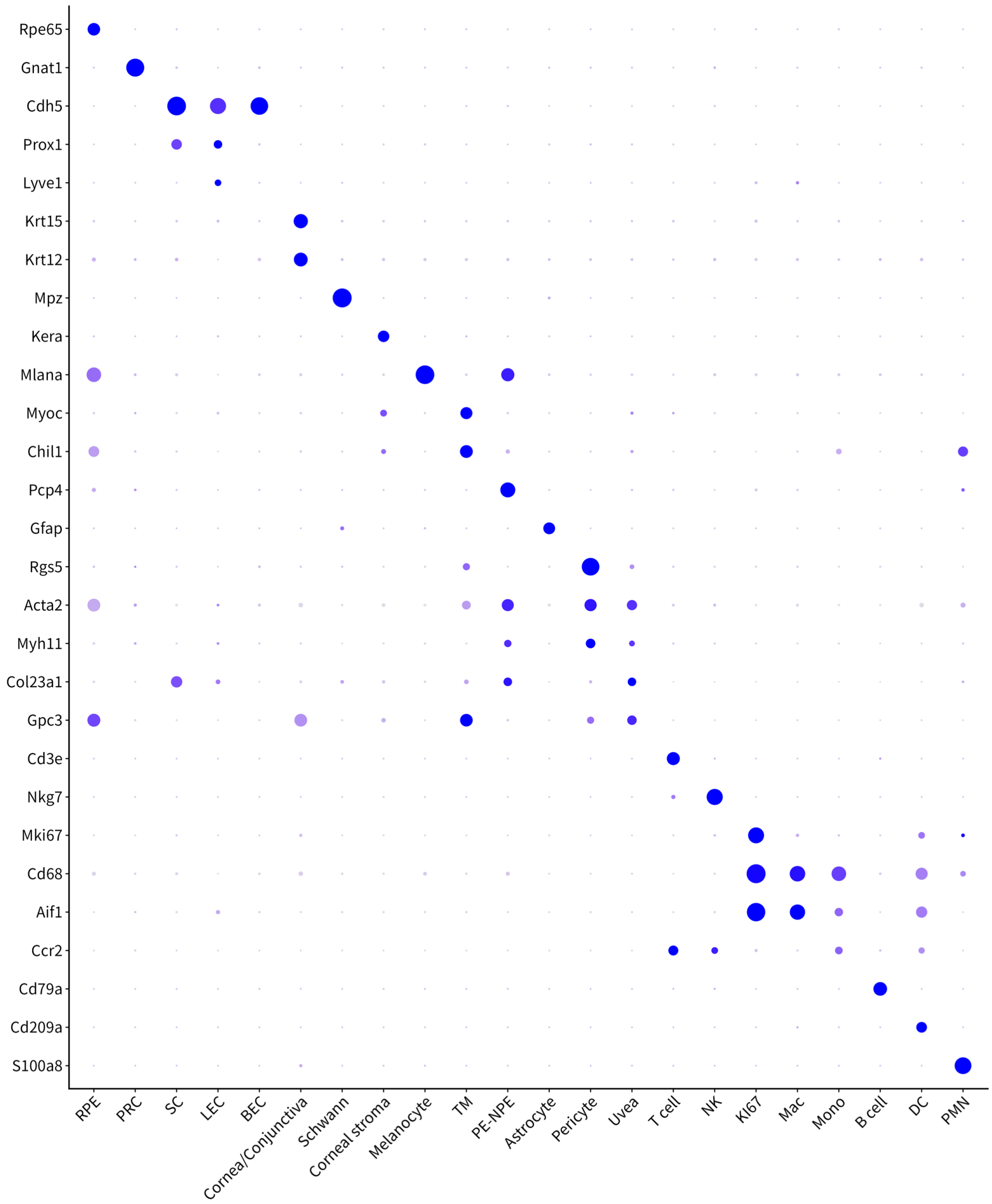

Supplementary Figure S1. Representative marker genes used for annotation of anterior segment cell populations in the integrated single-cell RNA-seq dataset.

Dot plot showing expression of selected canonical marker genes across the

major cell clusters identified in limbal/iridocorneal angle tissues. These markers support the cell-type annotations shown in Figure 1B. Dot size indicates the percentage of cells expressing each gene, and dot color indicates average expression level.

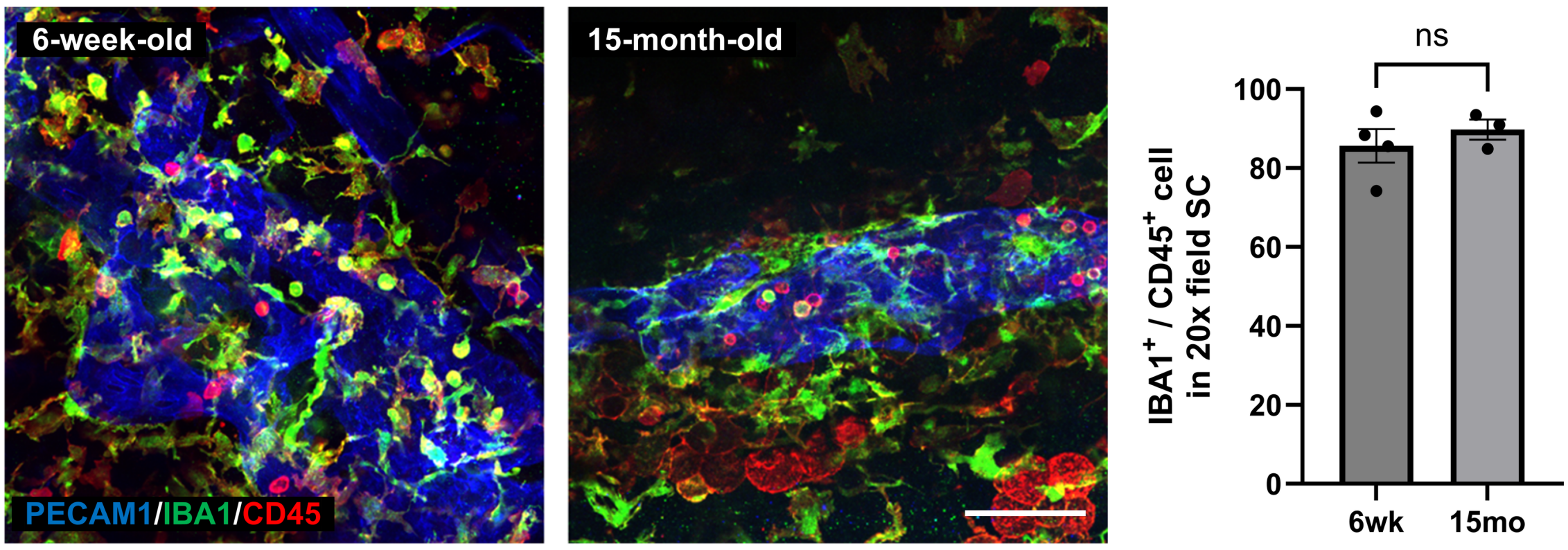

Supplementary Figure S2. The majority of CD45^{+} leukocytes in the Schlemm's canal region are IBA1^{+} at both young and older time points.

Representative PECAM1/IBA1/CD45 immunostaining images of the Schlemm's canal (SC) region showing overlap between IBA1 and CD45 signals in peri-SC immune cells. Quantification of the proportion of CD45^{+} cells that were IBA1^{+} in the SC region showed no significant difference between 6-week-old and 15-month-old mice. Scale bar, 100 μm. Data are presented as mean ± SEM. Student's two-tailed unpaired t-test was used. ns, not significant.

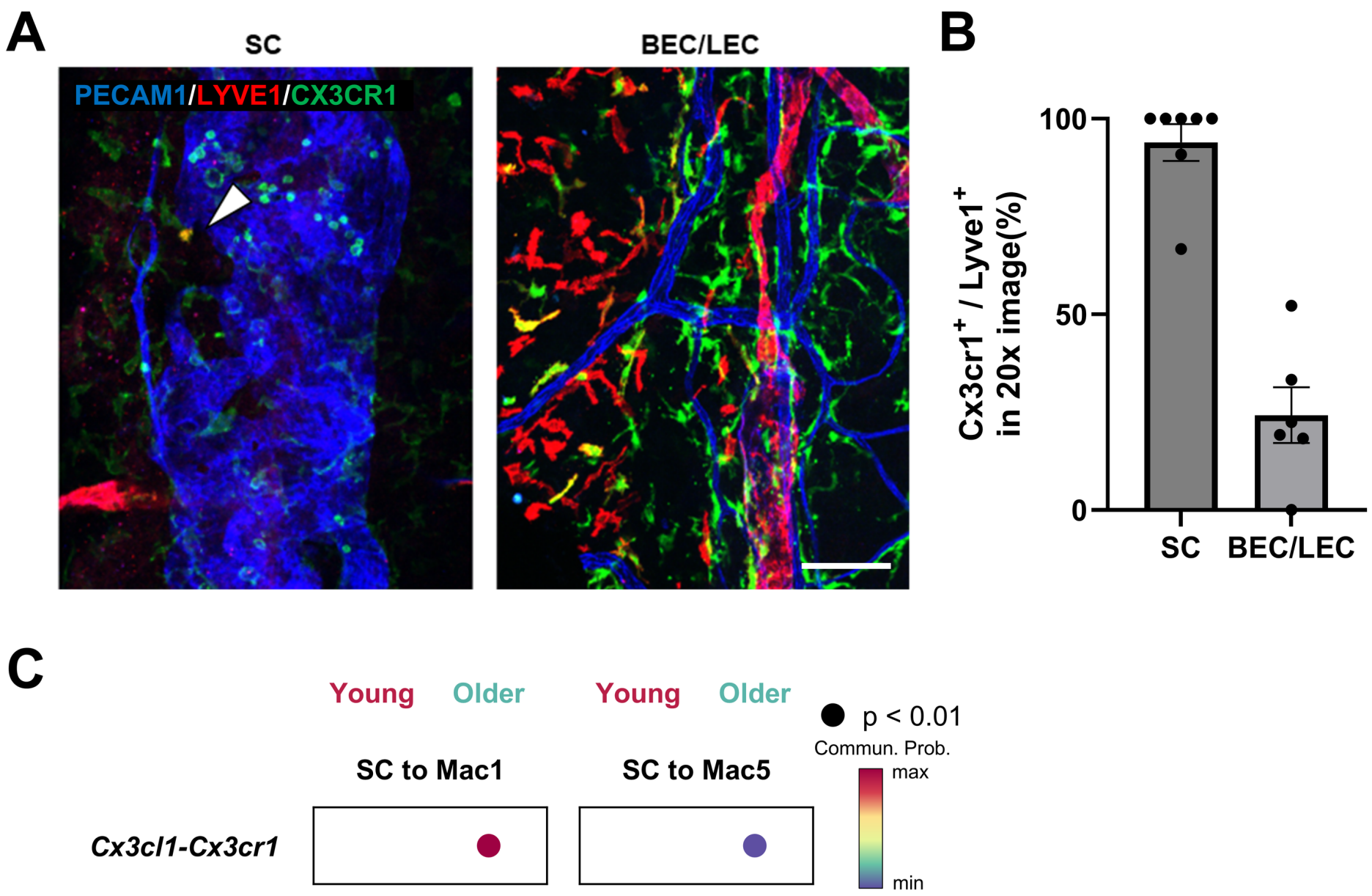

Supplementary Figure S3. CX3CR1 and LYVE1 expression patterns in macrophages across the conventional outflow pathway.

(A) Representative PECAM1/LYVE1/CX3CR1 immunostaining images of the Schlemm's canal (SC) region and of blood endothelial cell/lymphatic endothelial cell (BEC/LEC) regions. In the SC region, LYVE1$^+$ cells were rare, but when present they were predominantly CX3CR1$^+$ (arrowhead). In contrast, LYVE1$^+$ cells in the BEC/LEC region showed greater heterogeneity in CX3CR1 expression. Scale bar, 100 μm. (B) Quantification of the proportion of LYVE1$^+$ cells that were CX3CR1$^+$ in the indicated regions. Each dot represents one image. Statistical analysis was performed using a two-tailed unpaired Student's t-test. $P < 0.0001$. Data are presented as mean ± SEM. (C) Predicted *Cx3cl1–Cx3cr1* signaling between Mac1/Mac5 and SC in young versus older eyes (color, probability; black circle, $P < 0.01$).

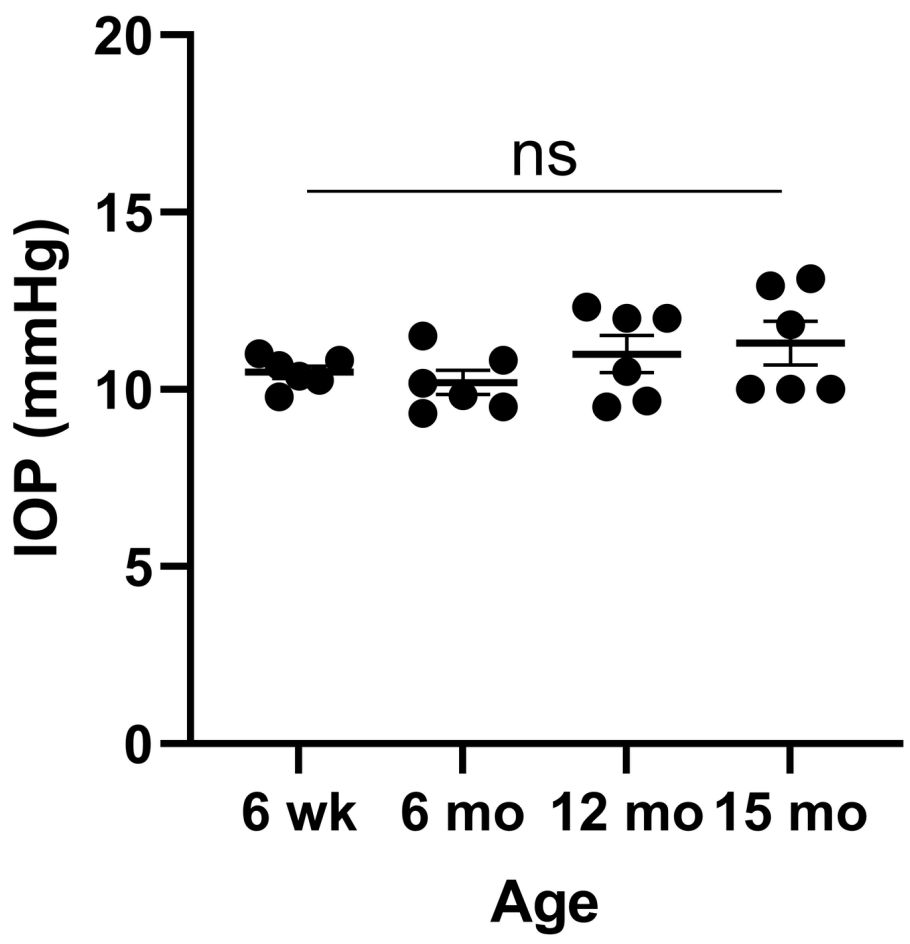

Supplementary Figure S4. Intraocular pressure does not show a significant age-dependent change in mice.

Intraocular pressure (IOP) was measured in mice at 6 weeks, 6 months, 12 months, and 15 months of age. No significant difference was detected among age groups. Data are presented as mean ± SEM. One-way ANOVA was used. ns, not significant.

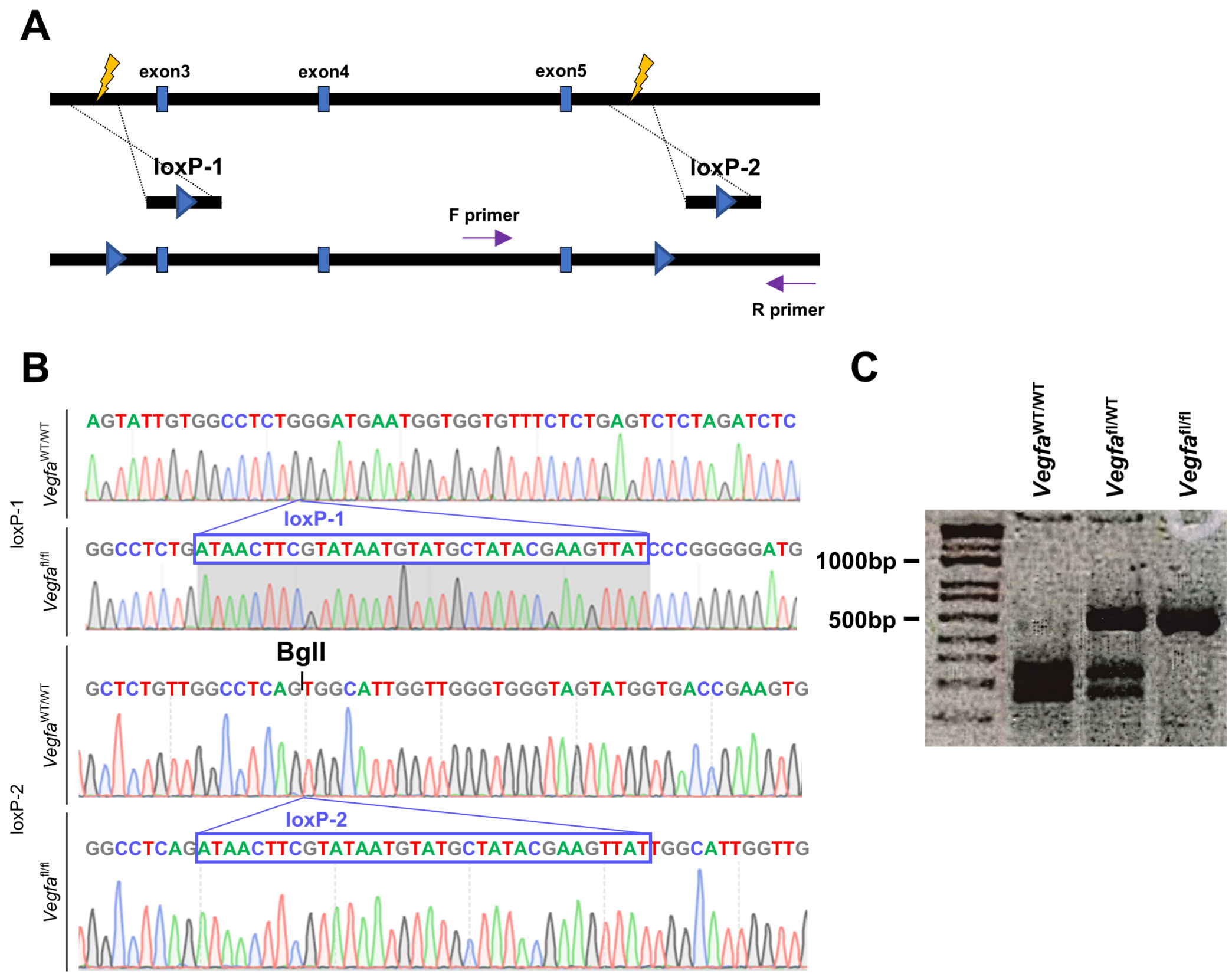

Supplementary Figure S5. Generation and validation of the *Vegfa* floxed allele.

(A) Schematic of the CRISPR-Cas9 targeting strategy used to introduce loxP sites into intron 2 (loxP-1) and intron 5 (loxP-2) of the *Vegfa* locus, thereby flanking exons 3–5. Purple arrows indicate the primers used for genotyping PCR. (B) Representative Sanger sequencing chromatograms confirming correct insertion of loxP-1 and loxP-2 in the *Vegfa* floxed allele. The inserted loxP sequences are boxed. (C) Representative PCR/BglI-based genotyping of *Vegfa* alleles. After BglI digestion, the wild-type allele yielded 305-bp and

247-bp fragments, whereas the floxed allele remained undigested at 587 bp; heterozygous mice showed both patterns. Lane order is *Vegfa*$^{wt/wt}$, *Vegfa*$^{wt/fl}$, and *Vegfa*$^{fl/fl}$.

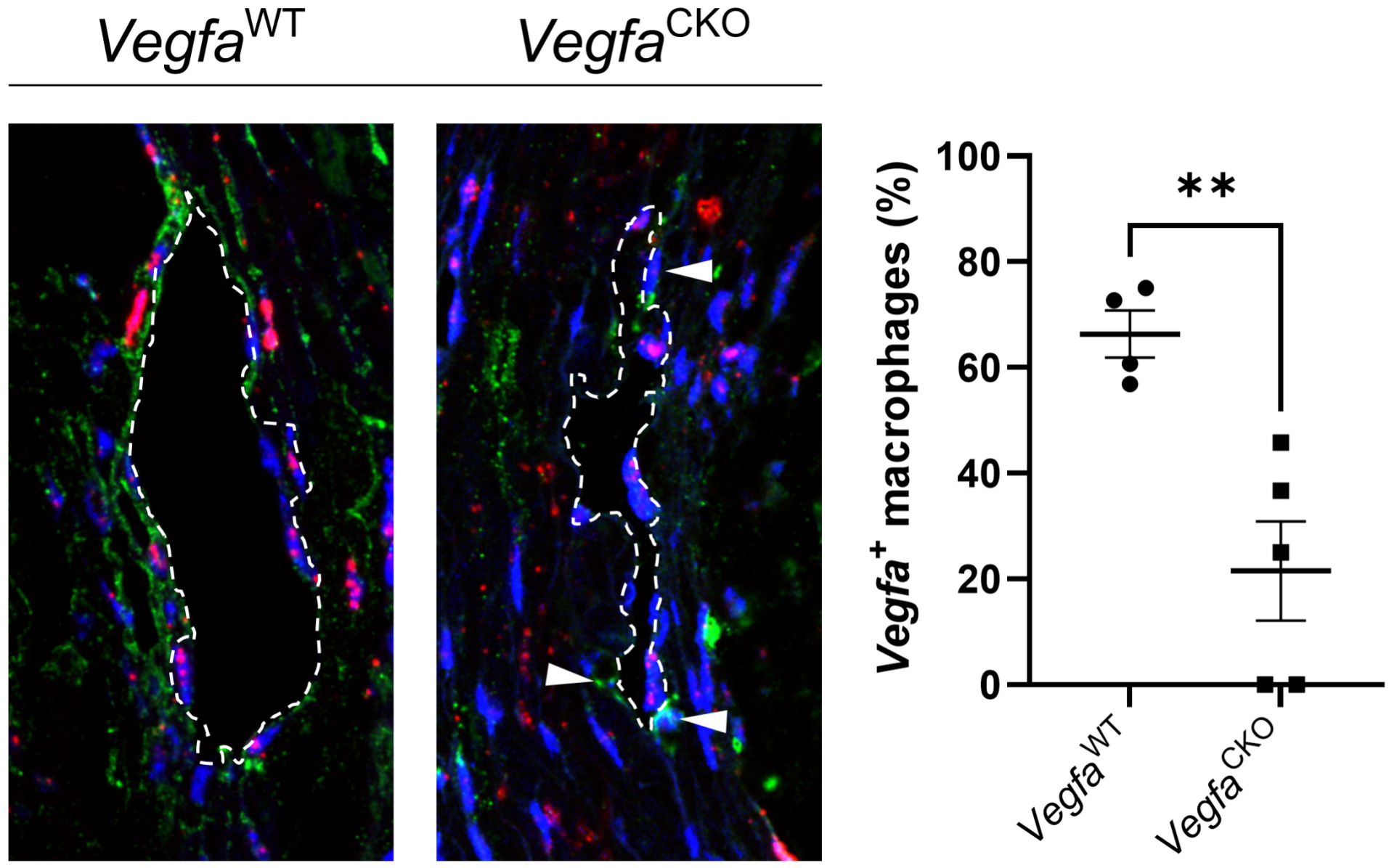

Supplementary Figure S6. *Vegfa* expression is reduced in Cx3cr1⁺ macrophages in 9-month-old *Vegfa* CKO eyes.

Representative *Vegfa* RNAscope (red) combined with IBA1 immunostaining (green) and DAPI (blue) images of the Schlemm's canal (SC) region from *Vegfa* WT and *Vegfa* CKO mice harvested at 9 months of age. Dashed lines outline the SC region. Arrowheads indicate peri-SC IBA1⁺ macrophages. Quantification shows the percentage of *Vegfa*-expressing macrophages among peri-SC IBA1⁺ macrophages around the SC. Data are presented as mean ± SEM. Each dot represents a mouse-level value generated by averaging image-level measurements where applicable (WT n = 4, CKO n = 5). Student's two-tailed unpaired t-test was used. **P < 0.01.

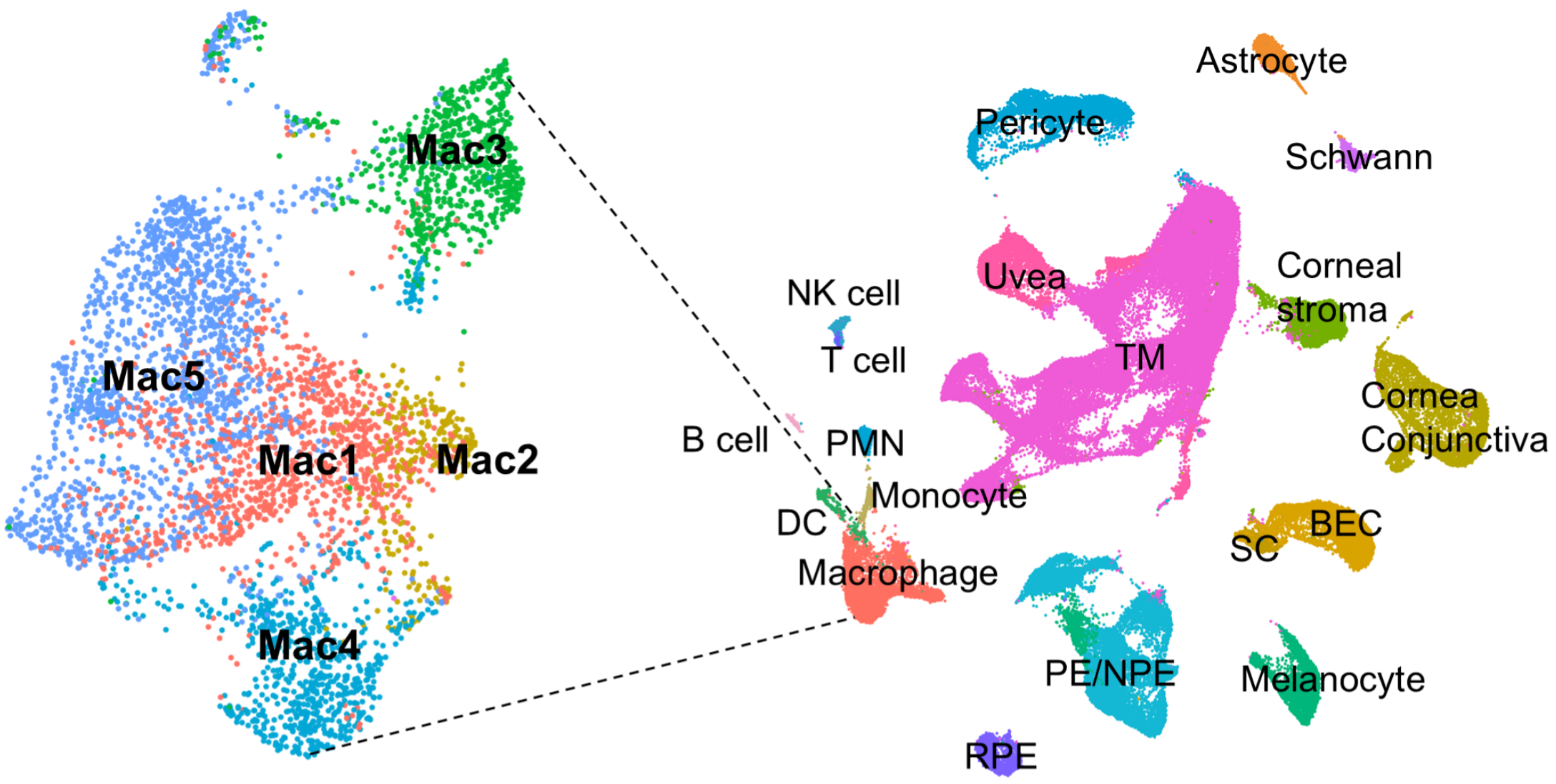

Supplementary Figure S7. Reference-guided annotation of myeloid populations in the *Tie2* dataset identifies monocytes and five macrophage subclusters.

UMAP of the integrated *Tie2* anterior segment dataset showing major cell populations (right) and magnified view of the myeloid compartment (left). Reference-guided transfer annotation based on the aging dataset resolved monocytes (Mono) and five macrophage subclusters (Mac1–Mac5), enabling cross-dataset comparison of myeloid cell states. Dashed lines indicate the myeloid compartment selected for magnified visualization.

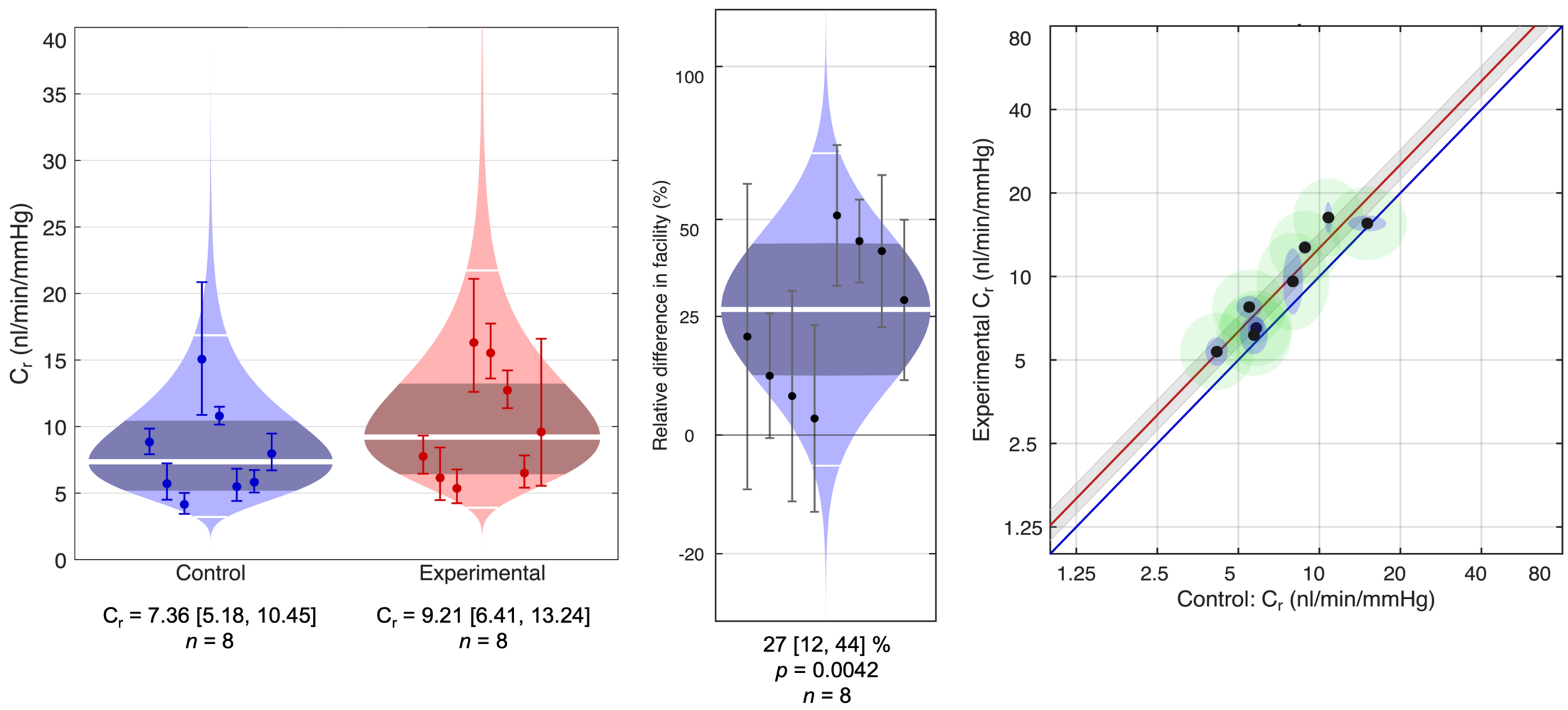

Supplementary Figure S8. Acute Hepta-ANGPT1 treatment increases conventional outflow facility in paired ex vivo eye perfusion.

Eyes from male C57BL/6J mice were analyzed in a paired design, with one eye perfused with Hepta-ANGPT1 and the contralateral eye perfused with Dulbecco's phosphate-buffered saline with calcium and magnesium, supplemented with 5.5 mM D-glucose (DBG) alone. Left, absolute conventional outflow facility (Cr) values for control and Hepta-ANGPT1-treated eyes. White lines indicate geometric means and dark shaded areas indicate 95% confidence intervals. Middle, paired relative difference in facility, showing a 27% increase in treated eyes relative to contralateral controls (95% CI, 12–44%; P = 0.0042; n = 8). Right, scatter plot of treated versus control Cr values. The red line indicates the fitted relationship with its 95% confidence interval (gray), and the blue line

indicates the line of identity. All Hepta-ANGPT1-treated eyes exhibited higher Cr than their paired control eyes. Statistical analysis of the paired relative difference was performed using the weighted paired t-test implemented in iPerfusion, which accounts for uncertainty in the fitted Cr estimate for each eye.

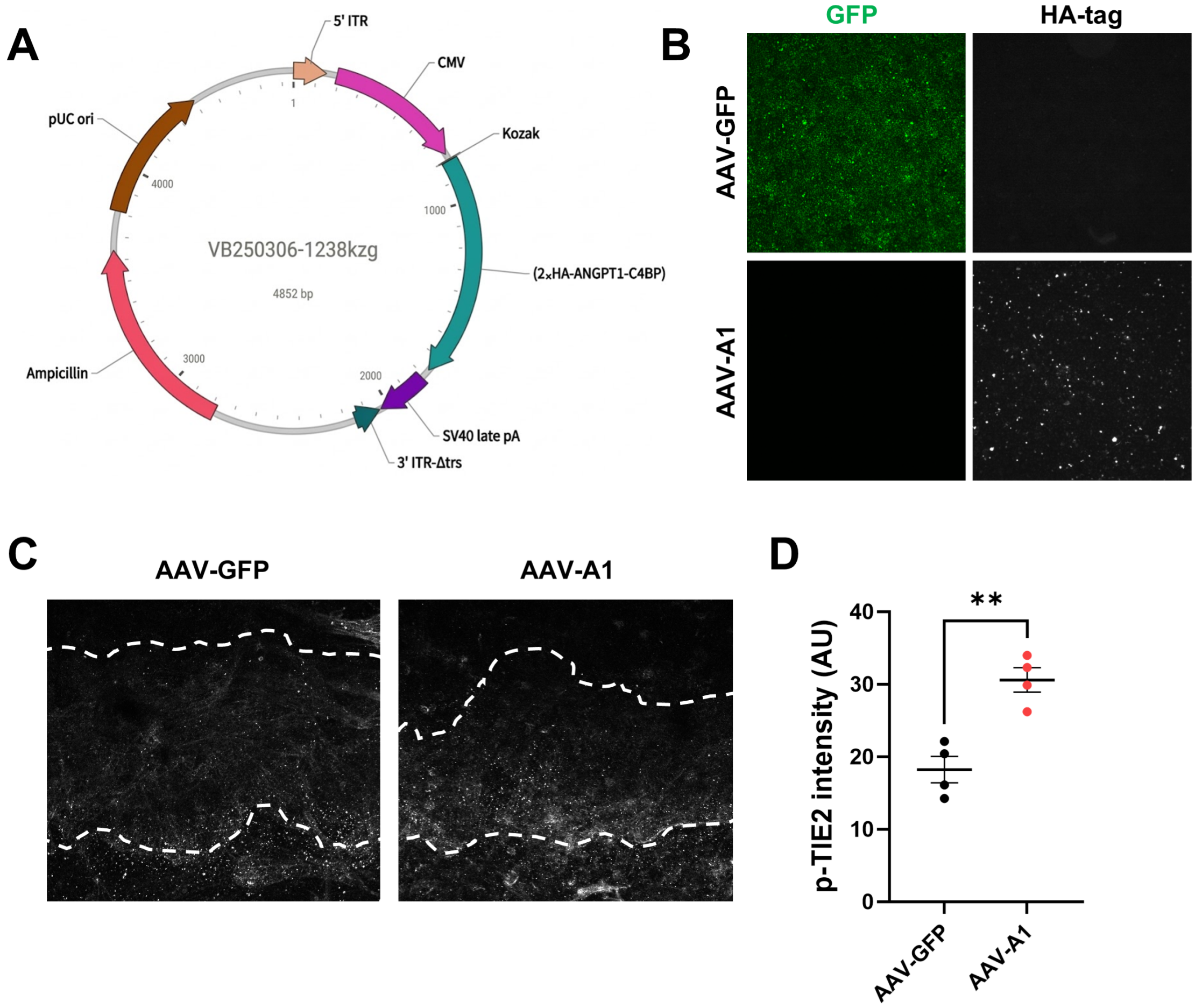

Supplementary Figure S9. Design, in vitro validation, and in vivo p-TIE2 activation by AAV-heptaAng1.

(A) Schematic of the self-complementary AAV expression construct encoding 2×HA-ANGPT1-C4BP under the control of the CMV promoter. (B) Representative fluorescence images of HEK293 cells exposed to control AAV-GFP or AAV-heptaAng1 (AAV-A1). GFP signal was detected only in the AAV-GFP condition, whereas HA-tag signal was detected only in the AAV-A1 condition, confirming in vitro expression of the AAV-encoded 2×HA-tagged

ANGPT1-C4BP transgene. (C) Representative p-TIE2 immunostaining images of the Schlemm's canal (SC) region from older mice treated with AAV-GFP or AAV-A1. Dashed lines outline the SC region. (D) Quantification of p-TIE2 intensity in the SC region, showing increased p-TIE2 in AAV-A1-treated eyes. Data are presented as mean ± SEM. Each dot represents one eye. Two-tailed unpaired Student's t-test was used. $^{**}P < 0.01$.

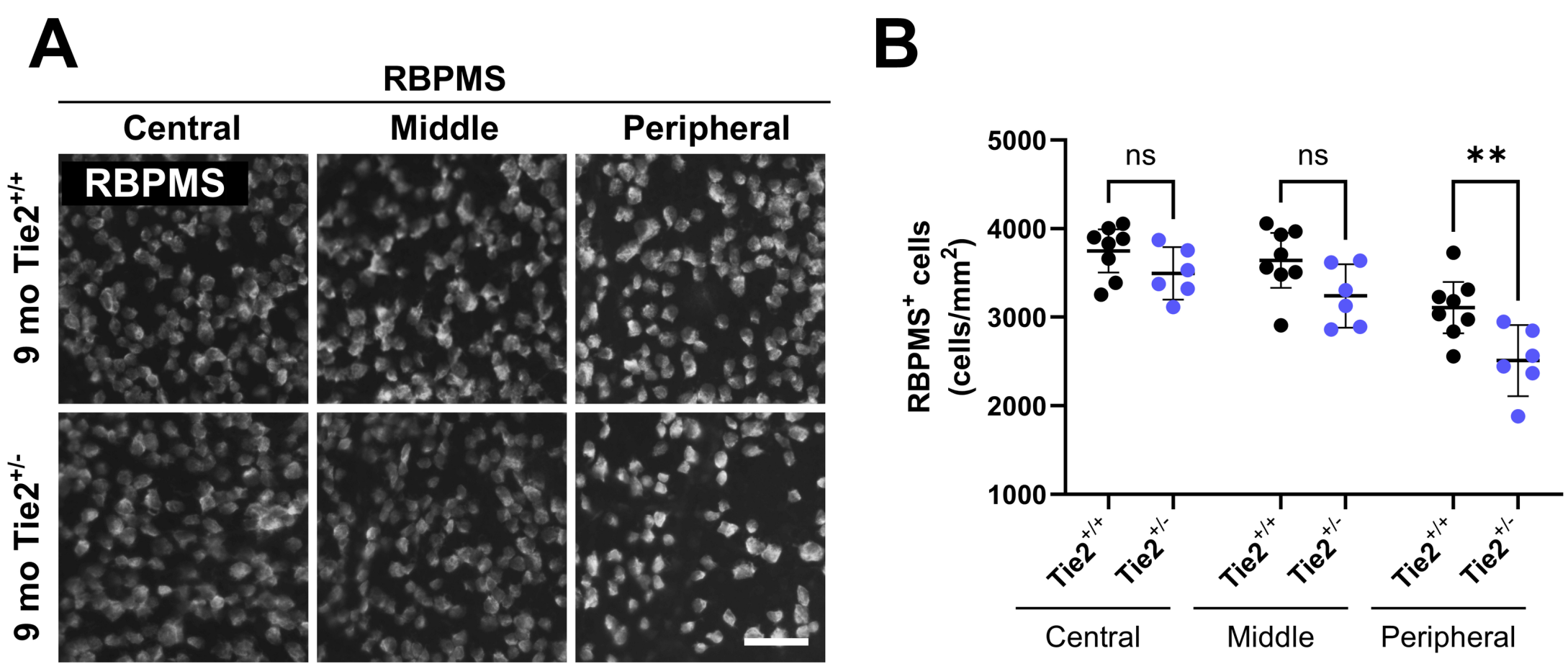

Supplementary Figure S10. ***Tie2*** **haploinsufficiency reduces peripheral retinal ganglion cell density at 9 months.**

(A) Representative RBPMS immunostaining images from central, middle, and peripheral retinal regions of 9-month-old *Tie2*$^{+/+}$ and *Tie2*$^{+/-}$ mice. Scale bar, 50 µm. (B) Quantification of RBPMS^{+} retinal ganglion cell density in the indicated retinal regions. Data are presented as mean ± SEM. Two-tailed unpaired Student's t-tests were used for each retinal region. ns, not significant; $^{**}P <$ 0.01.

Supplementary Table S1.

Human donor age and death-to-preservation interval

Age	Death-to-preservation
17	6 h 44 min
29	1 h 01 min
48	11 h 07 min
65	13 h 44 min
67	6 h 10 min
68	11 h 23 min
74	10 h 17 min
72	13 h 59 min

Supplementary Table S2.

Human donor age and death-to-preservation interval

Age	Death-to-preservation
48	10 h 25 min
72	13 h 59 min
58	13 h 12 min
41	4 h 57 min
24	7 h 38 min
48	9 h 35 min
40	13 h 55 min
62	15 h 05 min

Supplementary Table S3.

ssODN sequences used for loxP insertion at the *Vegfa* locus

Name	Sequence (5′–3′)
*Vegfa*_loxP_ssODN1	ggactggcaggtgtctctcgaagcttctgtgtctgtgcatgatatcagtgtcagaaggcatagccaacatagctc cttctaactagttcttactagtattgtggcctctgATAACTTCGTATAATGTATGCTATACGAAGT TATcccgggggatgaatggtggtgtttctctgagtctctagatctccat
*Vegfa*_loxP_ssODN2	actagcgtccacagcagagtgcaggagagcgagtgggtgccacagATAACTTCGTATAATGTAT GCTATACGAAGTTATcccgggggcagtttctgtgtatcaggggatggacgtagcctgggccttgttggg tggtgactgtggccatccaggttttttaggtcccaggagatttcttggttgtgtgtgtatgcctttccgtagggctgtg

Lowercase letters indicate homology arms, and uppercase letters indicate the loxP sequence.

Supplementary Table S4.

Materials used in the present study

Category	Reagent / item	Company / source	Identifier
Primary antibody	Anti-PECAM-1 (CD31)	BD Biosciences	553370
Primary antibody	Anti-Iba1	Wako	019-19741
Primary antibody	Anti-CD45	R&D Systems	AF114-SP
Primary antibody	Anti-LYVE1	AngioBio / Labome	11-034
Primary antibody	Anti-CD68	Abcam	ab125212
Primary antibody	Anti-RBPMS	PhosphoSolutions	1832-RBPMS
Primary antibody	Anti-Phospho-TIE2	R&D Systems	AF2720
Primary antibody	Anti-HA	Cell Signaling Technology	3724S
RNAscope probe	Probe - Mm-*Vegfa*	ACD; Bio-Techne	312931
RNAscope probe	Probe - Hs-*VEGFA*	ACD; Bio-Techne	423161
